# Kinesiophobia in Stroke Patients, Multiple Sclerosis and Parkinson’s Disesase

**DOI:** 10.3390/diagnostics11050796

**Published:** 2021-04-28

**Authors:** Dagmara Wasiuk-Zowada, Andrzej Knapik, Justyna Szefler-Derela, Anna Brzęk, Ewa Krzystanek

**Affiliations:** 1 Department of Physiotherapy, School of Health Sciences in Katowice, Medical University of Silesia, 40-754 Katowice, Poland; dwasiuk@sum.edu.pl (D.W.-Z.); jszefler@sum.edu.pl (J.S.-D.); 2 Department of Adapted Physical Activity and Sport, School of Health Sciences in Katowice, Medical University of Silesia, 40-754 Katowice, Poland; aknapik@sum.edu.pl; 3 Department of Neurology, Faculty of Medical Sciences in Katowice, Medical University of Silesia, 40-754 Katowice, Poland; ekrzystanek@sum.edu.pl

**Keywords:** kinesiophobia, multiple sclerosis, physical activity

## Abstract

Background: Stroke (S), multiple sclerosis (MS), Parkinson’s disease (PD) are chronic neurological diseases that are a challange for public health and represent a real social problem. Physical activity (PA) improves functional performance, reduces various symptoms in PD and MS, in stroke- reduced neurological impairment of patients and provides a chance for independence. One of the main obstacles in successful rehabilitation is patients’ movement passivity. The reason might be the psychological aspects, in particular fear of movement—kinesiophobia. *Aim*: To determine how many patients with S, MS, and PD suffer from kinsiophobia and what factors influence this process. Methods: Fifty patients after stroke, eighty one MS patients and sixty one PD patients were consecutively recruited from hospital and outpatients clinics. The sociodemographic data, self- assesment of fitness, Visual Analogue Scale (VAS) for pain, Tampa Scale of Kinesiophobia (TSK) and The Modified Baecke Questionnarie for Older Adults for physical activity were collected. A score >37 was considered to indicate a high level of kinesiophobia according to the TSK. Results: High level of kinesiophobia was shown in 66.67% of the subjects. TSK medians in particular illnesses were above the cut-off score and amounted: S—42.50 points; MS—38 points; PD—42.00 points. Regression showed 15% of fluctuation of variance (R2 = 0.1498; *p* < 0.0001), where regression factor showed: for mobility self-assessment: *b* = −0.2137 and for the age *b* = 0.0065. Conclusions: Kinesiophobia among the patients suffering from S, MS and PD concerns most of the subjects. Predictors of kinesiophobia are: limitations connected with functioning and age. The meaning of kinesiophobia in neurological disorders requires further research.

## 1. Introduction

Neurological diseases like stroke (S), multiple sclerosis (MS), Parkinson’s disease (PD) are not only a challenge for medical care but also a huge social issue. Stroke incidence rates are presented in statistics among other cardiovascular diseases (CVD). They speak of 24.3 million cases in 2016 [1]. In the case of MS, it is estimated that more than 2 million people suffer from this disease [2]. The frequency of PD increases with age–among people who are older than 60 years old, 1% of the population suffers from PD [3]. The epidemiological data shown points out that the scale of those illnesses is serious. Every year there are more patients suffering from cardiovascular diseases, which are caused by the aging of society. These diseases (S, MS, PD) have different causes and courses, but they are linked by limitation of mobility and the need to maintain functional fitness at the highest possible level (MS, PD) or return to it (S) for as long as possible.

The strong meaning of physical activity (PA) can be found in the literature which is necessary for minimalizing the symptoms of neurological diseases and in case of strokes—the chance for better functioning [4,5,6]. The main problem of successful rehabilitation and everyday life is the mobility inaction among patients. Apart from other factors like pain and tiredness [7,8,9], the reason for inaction of mobility can also be psychological factors–fear of movement—kinesiophobia. A biomedical understanding of kinesiophobia was presented by Kori et al. [10] assuming that the cause of the problem is the fear that physical activity will increase pain and/or disease symptoms. Another perspective on the understanding of this term was presented by Knapik et al. [11] who defined kinesiophobia as the fear of experiencing physical or psychological discomfort. Authors also noted that in patients characterized by kinesiophobia, there is a certain dissonance between the individual’s real abilities and their internal conception of them. The problem of kinesiophobia among neurological patients is not well known. Getting to know the scale of this problem and the conditions in which it appears should be taken into account in developing new or modifying already existing rehabilitation programmes.

The aim of this project was to investigate the prevalence and severity of kinesiophobia among patients with S, MS and PD. It was felt that its association with age of the subjects, severity and duration of symptoms, mobilities, pain and PA should also be investigated.

## 2. Materials and Methods

### 2.1. Participants

The choice of examination was intentional—patients suffering from S, MS and PD, from the neurological ward and neurological clinic of the university hospital, were enlisted. After informing the subjects about the purpose and the methods of the research and gaining their written consent, mental ability of the patients was checked. Test eligibility criterion was >24 points of Mini-Mental State Examination—MMSE [12].

A total of 192 people were examined—116 women (60.42%) and 76 men (39.58%). The age of the subjects—median 59.00; average number 58.61 (SD = 15.02); (±95% CI: 56.48–60.75) years.

The conditions for participation in the study were the recognition of:

- Stroke—according to WHO definition [13] (*n* = 50; 26.04% of all). This group included 27 women (54%) and 23 men (46%). Functional criterion: II or III degree according to the Rankin scale [14]. Due to meeting the condition of stabilization of the general condition, the subjects were examined in, at least, during fifth day after the stroke occurrence.
- Multiple sclerosis—according to the Mc Donald criterion [15] (*n* = 81: 42.19%). This group included 60 women (74%) and 23 men (26%). The criterion of the disease advancement according to the Expanded Disability Status Scale (EDSS) [16] win the range of 0–6.5 points. The subjcts were arbitrarily divided into three groups: I—EDSS < 2.5 pkt; II—EDSS 2.5–4.0 points; III—EDSS 4.5–6.5 points.
- Parkinson’s disease—according to the criterion of the UK Parkinson’s Disease Society Brain Bank [17]. Patients included had from I to III stage of the disease according to the Hoehn-Yahr scale (HYS) [18]. Number of the patients: *n* = 61 (31.77% of all). This group included 29 women (48%) and 23 men (52%).

### 2.2. Methods

The study used a questionnaire consisting of several parts. It consisted of the metrics part—gender, age, duration of illness, mobility self-assessment data were gathered. (1: person dependent, 2: partially independent, 3: fully independent) and the assessment of the most frequenly appearing pain according to the Visual Analogue Scale (VAS): 0–10, 0—no pain, 10—maximum pain [19].

To examine the level of kinesiophobia the linguistically adapted and in terms of examinated diseases Tampa Scale of Kinesiophobia (TSK) was used [10]. TSK consists of 17 statements, which have the answers assigned to them. These responses are scoring on a Likert scale from 1 (strongly disagree) to 4 (strongly agree). The scoring of questions 4, 8, 12, and 16 is reversed. The total score is in the range of 17 to 68 points—the more points—the higher level of kinesiophobia. Quality assessment of TSK was also included (no kinesiophobia or its intensification). The criterion accepted in the literature is that a score above 37 points. (cut-off point) indicates a high level of kinesiophobia [20,21]. Internal consistency which was initially tested of TSK was satisfactory: Cronbach’s α = 0.71.

Taking into consideration the age and the functional state, PA was examined with the use of The Modified Baecke Questionnarie for Older Adults [22,23]. This is a tool used for estimating the annual physical activity based on the self-description of the respondents. The type of activities performer is taken into consideration (intensity of effort), its weekly exercise time and the number of months during which the effort is made. Appropriate scoring allows the calculation of activity indicators. The total score, i.e., the sum of physical activity in three areas, was taken into consideration: household duties, sport and recreational activities [24].

### 2.3. Ethics Approval

The study was approved by the Bioethics Committee of the Medical University of Silesia in Katowice (Decision no. KNW/0022/KB/119/18; 19 June 2018 and PCN/0022/KB1/120/19; 12 November 2019). It is conformed to the Helsinki Declaration. Written consent was obtained from all the individual participants included in the study.

### 2.4. Statistical Analysis

Means and confidence intervals were calculated for the means (±95% CI) of the study variables: age of subjects, symptom severity, duration, self-assessment of fitness and PA. The Mann-Whitney U test was used to compare TSK-by gender. Comparison of TSKs—by disease type was performed using the Kruskal-Wallis ANOVA test. Kinesiophobia variability by functional level was calculated using the chi2 test. Correlations were calculated using Pearson’s correlation coefficients. The influence of the analyzed independent variables (age, duration of the disease, self-assessment of fitness, pain and PA) on the dependent variable—kinesiophobia was assessed using backward linear regression. The level of significance adopted: *p* < 0.05.

## 3. Results

The age of the subjects with S and PD was similar, and the medians and min—max values were: S—69.50, 35–92 years respectively; PD—68, 41–84 years old. People with MS were younger: median—43.00, min—max 35–69 years (ANOVA by Kruskal-Wallis: *p* = 0.000. Post-hoc: S–MS: *p* < 0.001; PD–MS: *p* < 0.0001). Given the duration of the disease, these values were: S-median—0.05, min–max—0.01–9.00 years; MS-median—9.00, min–max. 33–32.00 years; PD-median 8.00, min–max. 5–24.00 years (ANOVA by Kruskal-Wallis: *p* = 0.000. Post-hoc: S–MS: *p* < 0.0000; S-PD: *p* < 0.0000).

Self-assessment of physical fitness 1–2–3 was most favorable in the MS group: 1.23–20.99–77.78%. In other groups they were: S 8.00–62.00–30%; PD 8.20–55.74–36.06%. Taking into account the medians, patients with PD suffered the most: 5.00 points on the VAS scale, then MS: 4.00 points and S: 3.00 points.

**Figure 1:**
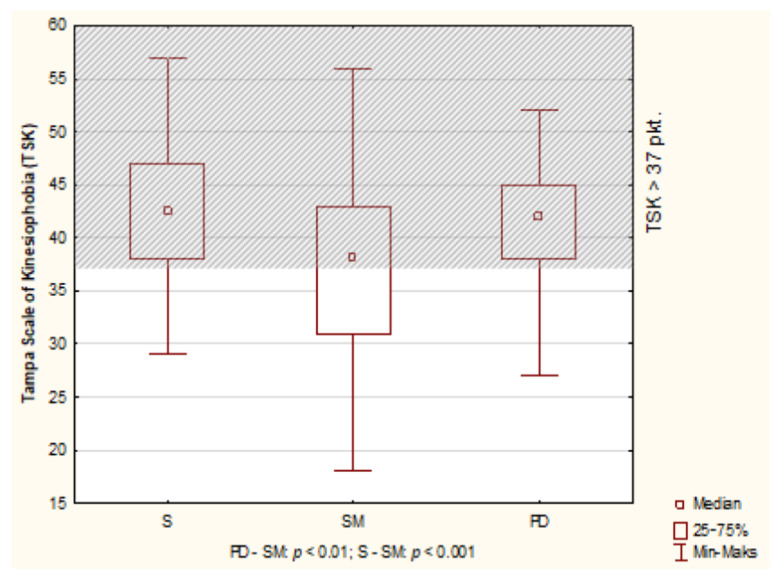
Results of Tampa Scale of Kinesiophobia (TSK) in S, MS and PD patients.

High kinesiophobia was seen in 128 people (66.67%). The median of TSK for all respondents was 39.51 points. There were no TSK differences between men and women. The significance levels were respectively: *p* = 0.1231 (all respondents); S: *p* = 0.1989; MS: *p* = 0.3348; PD: *p* = 0.1487. Also, median TSK in individual diseases were above the cut-off point and amounted to: S—42.50 points; MS—38 points; PD—42.00 points Comparison (ANOVA by Kruskal-Wallis) showed differences: *p* < 0.001 (Figure 1).

**Figure 2:**
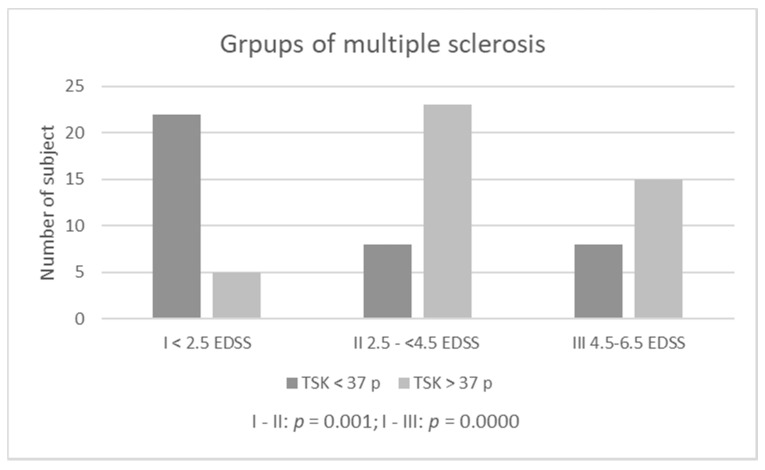
Kinesiophobia among multiple sclerosis patients according diability (EDSS).

Groups with the highest severity of kinesiophobia (>37 TSK points) are: S—Rankin II—people with a slight disability (83.33%) and PD-Stage III—people with balance disorders, mild or medium disability but independent (85.19%). The lowest percentage of people with kinesiophobia (>37 points) was among MS patients in group I: EDSS < 2.5 points. Considering the qualitative assessment of TSK (the presence or absence of kinesiophobia), a comparison of subjects according to scales determining the severity of disease symptoms (Rankin, EDSS, Hoehn-Yahr Scale) showed statistically significant differences only among those suffering from MS (Table 1, Figure 2), definitely people from group I had the lowest level of kinesiophobia.

It was considered that the size of the correlation coefficients of independent variables (age of the subjects, severity of symptoms, their duration, self-assessment of fitness and PA) (Table 2) allows to examine the full linear regression model, taking into account all analyzed variables. It was statistically significant: R2 = 0.17; *p* < 0.0001. However, sequential one-dimensional tests showed no significance (*p* > 0.05) for: duration of disease, pain and PA. This allowed the reduction of these variables and the construction of the final statistically significant model. It includes only two predictors: self-assessment of fitness and age, explaining 15% variance variation (R2 = 0.1498; *p* < 0.0001). Regression coefficients b (slope) were, respectively: for self-assessment of fitness: *b* = −0.2137 (*p* = 0.0000) and for age—*b* = 0.0065 (*p* = 0.0001).

## 4. Discussion

Originally, the term kinesiophobia referred to patients with spinal pain. The prevalence of this problem and its significance meant that kinesiophobia has been and continues to be studied in an increasing number of different disease entities [25,26,27,28,29]. For neurological diseases, there are few empirical studies devoted to kinesiophobia in neurological diseases [30,31,32] and this problem is still poorly understood.

A review of the literature found individual works carried out using TSK—people with PD [30], MS [31], S [32] as well as people with dystonia [33].

Descriptive statistics showed that the problem of kinesiophobia concerned patients suffering from all neurological diseases. The mean and median TSK scores were above the threshold considered high kinesiophobia—37 points, and this threshold exceeded by almost 67% of all respondents. This is an important observation. It justifies the thesis that the presence of a neurological disease (S, MS, PD) increases the risk of occurrence or exacerbating of kinesiophobia. It also confirms the need to diagnose this problem as early as possible in individual patients—before programming and implementing rehabilitation.

The analysis showed no differences in the occurrence of kinesiophobia between disease progression groups, neither among patients with PD nor after S. Differences were noted only in patients with MS. The reason for the differences in this group of patients was the relatively low percentage of patients diagnosed with kinesiophobia in group I (EDSS < 2.5 points). These were patients with a lack (0–1.5 points), slight or moderate disability [34]. In this group, central nervous system—CNS lesions do not yet interfere with daily activities, and gait function—considered a key motor function across the spectrum of disability among MS patients, it is not yet fully disturbed [34,35]. In addition, the subjects in this group had the lowest average age. The percentages of people with kinesiophobia in the other MS groups were high—comparable to the percentage of those with PD and S.

This observation underscores the association of functional abilities of neurologically ill individuals, and the self-assessments of these possibilities—as confirmed by the results of the presented study, with the occurrence of kinesiophobia. It also indicates the need for early rehabilitation and, if necessary, psychological support for the sufferers.

The authors believe that the patients with the highest percentage of kinesiophobia require separate comment. These were group III (Hoehn-Yahr)—PD and group II (Rankin)—S. In relation to patients with PD, such a high percentage seems to confirm the relationship between functional limitations and the occurrence of kinesiophobia. In stroke patients, a higher percentage of patients with kinesiophobia in Group II of the Rankin scale than in Group III can be explained by the significant influence of emotional factors, consisting of the relationship between the threatened sense of security and fear of movement [36]. While PD and MS are in the nature of a progressive process, stroke is sudden in nature, causing loss of multiple functions and a radical life change, which may translate into a high level of fear of movement. This has been confirmed in studies of patients after an acute coronary incident. They showed the highest level of kinesiophobia in the intensive care unit, which then fell (after 2 weeks) and reached the lowest level after 4 months [37]. This leads to the conclusion that the adaptation of patients after stroke should also take into account the gradual reduction of fear of movement—as appropriate health and functional options. This should be one of the key tasks of the therapeutic team. The presented regression results limited the independent variables included in this study to two statistically significant predictors of kinesiophobia: age and self-assessment of fitness. They explained a relatively small percentage of variance, which indicates the complexity of Kinesiophobia [11]. In addition to age and disease limitations, personality traits, pre-disease habits, the patient’s social situation, or psychological support or lack thereof may play a role. It should also be taken into account that due to the selection for the study, a significant proportion were elderly. Senior age is commonly associated with multiple diseases [38], often limiting the functional possibilities and strengthening the servo mechanism: movement possibilities—passivity-kinesiophobia [39,40,41].

Therefore, the problem of kinesiophobia should be taken into account when planning exercises for prevention and geriatric rehabilitation [42]. What is more, the noticed favorable relationship between fitness self-assessment and a lower level of fear of movement [36] emphasizes the importance of psychological support for people who are ill during therapy.

The lack of association between kinesiophobia and the duration of the disease and pain noted in this presented study is consistent with the results of studies in other diseases [28,43]. An important observation is that pain not felt by patients, but rather fear of its potential occurrence leads to kinesiophobia. This suggests the individual nature of its occurrence and confirms the need to search for its determinants, also among non-biomedical factors. This thesis is confirmed by the associations of kinesiophobia with lack of expected treatment effects [44], quality of life, affective disorders [45], or kinesiophobic attitudes in medical personnel [43,46].

The very weak correlation of TSK with PA may be explained by the generally low level and low variety of activity. This suggests further research using various research tools. It also suggests a systemic study of kinesiophobia and PA of patients hospitalized during medical check-up or physiotherapy visit. Patients with high levels of kinesiophobia should receive special comprehensive care in terms of promoting PA, regarding both direct rehabilitation and habitual outpatient care. Telemedicine, which, through a smartphone app, is able to coordinate the process of physical activity while being supervised by a physician or physiotherapist, can be a helpful tool in this effort [47]. In the opinion of the authors of this study, solutions based on new technologies are of great importance in the process of continuous monitoring of the progress and effects of treatment, including physiotherapy. Because of the threats connected with SARS-CoV2 pandemic it is especially important for less fit patients although it may also present some handling problems for elderly patients.

### Limitations

The study undertaken in order to initially recognize the scale and its determinants entails limitations typical of this type. The limitations of the current study include its cross-sectional design and cohort size and the fact that they relate to both the selection of respondents and organizational possibilities to reach respondents. 

The choice of PA questionnaire may also be debatable. The original version of the Baecke questionnaire included questions about the subjects’ work activity. The modified version that was used in the study does not include this component. The rationale for this choice was that due to the age and illness of the subjects, the vast majority of the respondents were economically inactive. Future studies should not only be carried out on larger scales but also take into account other factors connected to. It is the authors’ belief that in the didactic process of physiotherapists, neurologists, the emphasis should be on education regarding the sphere of the kinesiophobia problem.

## 5. Conclusions

The problem of kinesiophobia among people with S, MS and PD is widespread, affecting the vast majority of patients. The duration of the disease and the intensity of the pain are not related to kinesiophobia. Its predictors—self-assessment of performance and age affect it only to some extent, which points to the causes of this problem in the psychological rather than biological sphere.

The importance of the problem of kinesiophobia among neurologically ill patients requires further research into not only its determinants and impact on the therapy process, but also ways of reducing this personality trait.

## Figures and Tables

**Table 1:** Means and confidence intervals (±95%) of the studied variables and differences due to the severity of symptoms among S, MS and PD patients.

Variable		Age (Years)	Duration of Illness (Years)	Self-Assessment of Fitness (Point)	Pain (1–10)	PA (Point)	TSK (Point)	Kinesiophobia: TSK > 37 Point	
								% of Group	*p*
S *	II *n* = 24	69.63 64.24–75.01	0.25 0.00–0.55	1.54 1.33–1.76	2.87 1.75–4.00	3.47 2.08–4.86	41.46 38.78–44.13	83.33	nss
	III *n* = 26	70.15 64.83–75.48	0.98 0.09–1.87	2.00 1.77–2.23	2.88 1.79–3.98	2.57 1.05–4.10	42.85 39.98–45.71	73.08	
MS **	I *n* = 27	41.30 39.16–43.43	6.99 5.06–8.92	1.00	2.19 1.31–3.06	5.87 4.30–7.45	30.56 27.71–33.40	18.52	<0.0001
	II *n* = 31	46.29 43.38–49.20	9.82 7.66–11.97	1.26 1.07–1.45	4.35 3.53–5.18	5.13 3.30–6.95	39.19 36.89–41.50	74.19	
	III *n* = 23	49.39 44.94–53.84	14.43 10.72–18.15	1.48 1.26–1.70	4.17 3.24–5.11	3.97 2.77–5.16	40.91 37.65–44.18	65.22	
PD ***	I *n* = 8	71.13 67.07–75.18	7.31 1.17–13.46	1.63 1.00–2.25	4.88 2.41–7.34	5.72 1.83–9.61	40.88 34.58–47.17	62.50	nss
	II *n* = 26	63.42 59.50–67.35	7.33 5.62–9.03	1.54 1.30–1.77	5.19 4.43–5.95	8.06 4.90–11.21	39.88 37.62–42.14	69.23	
	III *n* = 27	68.70 65.45–71.96	10.20 7.85–12.56	1.93 1.71–2.14	5.11 4.49–5.73	3.01 1.61–4.42	41.93 39.55–44.30	85.19	

Abbreviations: nss, not statistically significant; PA, physical activity; TSK, Tampa Scale of Kinesiophobia; S, stroke; MS, multiple sclerosis; PD, Parkinson’s disease; * Rankin, ** EDSS, Expanded Disability Status Scale; *** HYS, Hoehn-Yahr Scale.

**Table 2:** Correlations of the studied variables: age, duration of the disease, self-assessment of fitness, pain, physical activity and kinesiophobia among the respondents in total.

Variable	Age	Duration of the Disease	Self-Assessment of Fitness	Pain	PA
Duration of the disease	−0.285 ***				
Self-assessment of fitness	−0.318 ***	nss			
Pain	nss	nss	−0.173 *		
PA	−0.218 **	nss	0.216 **	nss	
TSK	0.402 ***	nss	−0.366 ***	0.208 **	−0.255 ***

* *p* < 0.05; ** *p* < 0.01; *** *p* = 0.000; nss, not statistically significant; PA, physical activity; TSK, Tampa Scale of Kinesiophobia.

## Data Availability

Data are archived with the first author and in the Department of Neurology of the University Hospital where they were conducted. If necessary, contact the first author by e-mail at dwasiuk@sum.edu.pl or www.katedrafizjoterapii.sum.edu.pl, accessed on 28 Apirl 2021.
